# Severity of Ascending Aortic Degeneration in Patients With Chronic Obstructive Pulmonary Disease

**DOI:** 10.7759/cureus.90001

**Published:** 2025-08-13

**Authors:** Essi Pirskanen, Mona Laaksonen, Trina Chen, Ivana Kholova, Otso Arponen, Petteri Kauhanen, Aada Lattu, Timo Paavonen, Ari Mennander

**Affiliations:** 1 Department of Cardiothoracic Surgery, Tampere University, Tampere, FIN; 2 Department of Pathology, Fimlab Laboratories, Tampere University Hospital and Tampere University, Tampere, FIN; 3 Department of Radiology, Tampere University Hospital and Tampere University, Tampere, FIN; 4 Department of Clinical Radiology, Kuopio University Hospital, Clinical Imaging Center, Kuopio, FIN; 5 Department of Cardiothoracic Surgery, Tampere University Hospital, Heart Hospital, Tampere, FIN

**Keywords:** aortic dilatation, aortic surgery, ascending aortic degeneration, chronic obstructive pulmonary disease, histology

## Abstract

Background

Ascending aortic dilatation is associated with increased susceptibility to aortic events. Chronic obstructive pulmonary disease (COPD) may add to tissue degeneration and inflammation associated with the risk of increased aortic dilatation. We studied the characteristics and aortic wall degeneration of patients with COPD during ascending aortic dilatation.

Methodology

We enrolled 35 consecutive patients who underwent elective surgery for ascending aortic dilatation between February 2016 and November 2016. The patients were grouped according to the presence of COPD. The aortic diameters were measured for the aortic valve annulus, aortic root, sinotubular junction, ascending aorta, aortic arch, and descending aorta. An extensive analysis of ascending aortic wall histopathology was performed.

Results

The patients with chronic obstructive pulmonary disease (COPD) (n=7) all had aortic valve regurgitation, while the aortic wall had more mucoid extracellular matrix accumulation, medial degeneration, elastic fiber fragmentation and loss, and adventitial fibrosis than those without COPD (5.7±1.1 vs. 4.3±1.0 point score units (PSU), *p*=0.006; 2.7±0.5 vs. 1.7±0.8 PSU, *p*=0.006; 4.5±1.2 vs. 3.3±1.2 PSU, *p*=0.034; and 0.5±0.5 vs. 0.1±0.3 PSU, p=0.023, respectively). Only the distal ascending aortic diameter slightly differed in patients with COPD vs. those without (36.5±3.3 vs. 32.2±3.8 mm, p=0.011), although increased aortic dilatation was otherwise present in both groups.

Conclusions

We identified a subgroup of patients with COPD who had increased aortic wall degeneration during ascending aortic dilatation. Risk stratification of aortic events in patients with COPD warrants further clarification.

## Introduction

Ascending aortic dilatation is associated with increased susceptibility to acute aortic events [1]. Though several risk factors are identified during increased ascending aortic dilatation, the pathogenesis of aortic dilatation remains unclear [2]. Increased ascending aortic dilatation is often associated with the presence of a bicuspid aortic valve [3,4], and the presence of increased aortic root dilatation during aortic valve regurgitation has been described as the root phenotype [5]. Genetic background, including several connective tissue disorders such as Marfan syndrome and Loeys-Dietz syndrome, adds to the risk profile [6]. Many traditional cardiovascular risk factors, e.g., high blood pressure, male sex, and smoking, are associated with aortopathy leading to ascending aortic dilatation [5].

Many of these traditional patient characteristics are also present in patients with chronic obstructive pulmonary disease (COPD) [7,8]. Patients with COPD and emphysema often have enlargement of the ascending aorta [9]. COPD has been reported to increase the risk of ascending aortic dilatation and rupture [8]. COPD may also indirectly affect the aorta via increased tissue degeneration and inflammation [10]. Similar histopathological changes destroying both the lung parenchyma and causing aortic wall degeneration in patients with COPD may lead to increased weakening of the aortic wall and ascending aortic dilatation [9]. Understanding whether COPD contributes distinctly to aortic degeneration could significantly influence clinical strategies for monitoring and intervention, potentially reducing acute aortic complications.

We hypothesized that patients with COPD and ascending aortic dilatation exhibit distinctive histopathological changes in the aortic wall compared to those without COPD. Histological comparison of ascending aortic wall changes during aortic dilatation in patients with COPD versus those without has not yet been investigated. The aim of this study was to investigate the association of aortic wall degeneration in patients with or without COPD undergoing surgery for ascending aortic dilatation.

This article was previously presented as a meeting abstract at the 2024 Annual Meeting of the Scandinavian Association for Thoracic Surgery on September 12, 2024.

## Materials and methods

Study design and ethics 

The local institutional review board (Ethical Committee of the Tampere University Hospital, Tampere, Finland, R23028) and the Finnish Medicines Agency (Fimea FIMEA/2023/002785) approved this study, and the need for informed consent was waived due to the retrospective nature of the study. The study was conducted under the Declaration of Helsinki (as revised in 2013). All authors are accountable for all aspects of the work and ensure questions related to the accuracy and/or integrity of any part of the work. 

Patient selection and exclusion criteria

We thus enrolled 35 patients who underwent first-time elective surgery for asymptomatic aortic dilatation between February 2016 and November 2016. Patients with genetic aorta disease and dissection were excluded. 

COPD definition

COPD was defined as the presence of chronic bronchitis or emphysema and confirmed by the combination of the following ﬁndings: a history of heavy smoking, wheezing on auscultation, and patient self-reported experience of wheezing together with postbronchodilator lung function values of decreased ratio of forced expiratory volume in the first second to forced vital capacity on spirometry less than 0.70 [11].

Diagnostic criteria

Ascending aortic dilatation was preoperatively confirmed and evaluated with echocardiography. The severity of aortic valve regurgitation included moderate to severe regurgitation graded as 2 or 3 out of 3. Computer tomography (CT) imaging was performed prior to surgery according to routine clinical practice using one of the three CT scanners (Philips Brilliance Big Bore (Philips Healthcare, the Netherlands), Siemens SOMATOM Confidence 64 (Siemens Healthineers, Germany), or Toshiba Aquilion LB (Toshiba Medical System, Japan). The slice thickness was 3 mm. The scanning area encompassed the whole of the aorta.

Radiological assessments

The thoracic aortic diameters were re-measured on the hospital’s Picture Archiving and Communication Systems (PACS; Sectra vs. 24.2) in every patient from the following segments: the aortic annulus, the sinus of Valsalva, the sinotubular junction (STJ), the mid-ascending aorta, the distal ascending aorta, and the mid-descending thoracic aorta. Multiplanar reconstruction of the images allowed for three-dimensional presentation of the structures. We measured two perpendicular measurements from the segments, and the maximum aortic diameter was selected for comparison. Measurements were obtained by drawing lines from outer-to-outer vascular wall, vertical to the centerlines of the vessel. The thoracic aorta was considered dilated if its greatest dimension was >40 mm in any of the measured locations.

Surgical procedures

Surgery for dilatation of the thoracic aorta was considered if the maximum diameter of the aorta reached more than 55 mm or if an imminent aorta diameter growth of more than 0.3 mm per year was observed. The operating surgeon decided for the extension of the resection and surgical technique. When the aortic wall, including the sinotubular junction (STJ), was estimated as the reason for aortic regurgitation, STJ was prepared for a suitable graft in an aortic root-sparing fashion with/without aortic valve surgery (root-sparing surgery). A radical resection of the dilated ascending aorta, including the root and the aortic valve, was performed whenever needed. The aortic arch was resected completely or in a hemiarch fashion, depending on the involvement of aortic wall disease. The size of the graft was estimated at surgery. Since the surgical procedure was performed upon surgical decision, the sample was procured from the middle of the resected area of the ascending aorta in the vicinity of STJ. 

Histological and immunohistochemical evaluation

A minimum of six pieces of resected ascending aorta, including all three aortic wall layers, i.e., the intima, the media, and the adventitia, were embedded in paraffin, cut to 4 mm thick sections, and stained with hematoxylin and eosin, elastic Verhoeff-van Gieson, and periodic acid-Schiff-Alcian Blue. At least 18 sections (six stained with hematoxylin-eosin, six stained with Verhoeff-van Gieson stain, and six with periodic acid-Schiff-Alcian Blue) were evaluated in each case [4].

Immunohistochemistry was performed using the Ventana Lifesciences Benchmark XT© (Roche Diagnostics, Rotkreuz, Switzerland) staining module for T and B lymphocytes, plasma cells, macrophages, and smooth muscle cells. Ventana Lifesciences Antibody Dilution Buffer© (Roche Diagnostics, Rotkreuz, Switzerland) was utilized for dilution media. Immunohistochemistry was used to characterize the inflammatory infiltrate (CD3 as a marker for T-lymphocytes [clone 2GV6, RTU, Roche Oy, Espoo, Finland], CD68 as a macrophage marker [clone KP1, RTU, Roche Oy, Espoo, Finland], CD38 for plasma cells [clone SP149, RTU, Roche Oy, Espoo, Finland], alpha-smooth muscle actin for smooth muscle cells [clone 1A4, Sigma Biosciences, St. Louis, Missouri], and CD20 as a B-lymphocyte marker [B-lymphocytes (clone L26, RTU, Roche Oy, Espoo, Finland]). 

Hematoxylin-eosin was used for overall evaluation, inflammation assessment, and evaluation of smooth muscle cell nuclei. Verhoeff-van Gieson was applied in the assessment of elastic fibers, including laminal medial collapse. Periodic acid-Schiff-Alcian Blue was used for mucoid extracellular matrix accumulation assessment and fibrosis evaluation. 

Quantification of medial degeneration

The aortic specimens were assessed as a part of routine surgical pathology evaluation according to guidelines of The Society for Cardiovascular Pathology and The Association for European Cardiovascular Pathology [12] by two experienced cardiovascular pathologists (IK, TP). The assessed features included overall medial degeneration, mucoid extracellular matrix accumulation, elastic fiber loss/fragmentation, elastic fiber thinning, elastic fiber disorganization, smooth muscle cell nuclei loss, laminal medial collapse, smooth muscle cell disorganization, medial fibrosis, vasa vasorum medial thickening, and adventitial fibrosis. All features were graded for severity (absent, mild, moderate, and severe) and distribution (focal, multifocal, and extensive). The worst present grade/distribution was reported and semi-quantified on a scale of 0-3 [12]. The grade and distribution of the features were added to obtain a total semiquantitative and representative value expressed as point score units (PSU) [4]. 

Statistical analysis

Continuous variables were expressed as means with standard deviation (SD) and compared using the Mann-Whitney U test. Categorical variables were presented as numbers and percentages and were compared using the Chi-square (χ2) or Fisher’s exact tests, when n < 5. The patients were divided into those with or without COPD. The predictive value of the extent of mucoid extracellular matrix accumulation alone, representing the severity of aortic wall degeneration using a receiver operating characteristic (ROC) curve, was analyzed for the association in patients with vs. without COPD. All analyses were performed with IBM Corp. Released 2022. IBM SPSS Statistics for Windows, Version 28. Armonk, NY: IBM Corp., with p < 0.05 as the significance criterion.

## Results

Patient characteristics

A total of 35 patients were included in this retrospective study, of which seven (20.0%) had COPD. None of the patients had systemic immunosuppressive treatment. Mean age was 65 (10) years. Patients with COPD often had asthma (3 [42.9%] vs. 0, respectively, p < 0.001), had aortic valve regurgitation (7 [100.0%] vs. 16 [57.1%], respectively, p < 0.033), and none had bicuspid aortic valves (0 vs. 16 [57.1%], respectively, p < 0.007) as compared with those without. None of the patients had connective tissue disorder, vasculitis, or previous cardiac surgery (Table 1).

**Table 1:** Patient characteristics COPD: chronic obstructive pulmonary disease; n: number; SD: standard deviation; Continuous variables were expressed as means with SD and compared using the Mann-Whitney U test. Categorical variables were presented as numbers and percentages and were compared using the Chi-square (χ2) or Fisher’s exact tests, when n < 5. p < 0.05 was considered significant. *Test statistic values include z-values for the Mann-Whitney test.

	All patients	With COPD	Without COPD	Test statistic value	p-value
Number of patients (%)	35 (100.0)	7 (100.0)	28 (100.0)		
Age, years, mean (SD)	65 (10.2)	68 (8.6)	65 (10.6)	-0.66*	0.508
Sex				2.86	0.091
Female, n (%)	7 (20.0)	3 (42.9)	4 (14.3)		
Male, n (%)	28 (80.0)	4 (57.1)	24 (85.7)		
Hypertension, n (%)	25 (71.4)	5 (71.4)	20 (71.4)	0	>0.999
Asthma, n (%)	3 (8.6)	3 (42.9)	0	13.12	<0.001
Diabetes, n (%)	4 (11.4)	1 (14.3)	3 (10.7)	0.07	0.791
Arthritis, n (%)	1 (2.9)	0	1 (3.6)	0.26	0.612
Hypercholesterolemia, n (%)	11 (31.4)	2 (28.6)	9 (32.1)	0.03	0.856
Coronary artery disease, n (%)	8 (22.9)	0	8 (28.6)	2.59	0.107
Strokes, n (%)	4 (11.4)	0	4 (14.3)	1.13	0.288
Aortic valve insufficiency, n (%)	23 (65.7)	7 (100.0)	16 (57.1)	4.56	0.033
Bicuspid aortic valve, n (%)	16 (45.7)	0	16 (57.1)	7.37	0.007
Maximal aortic diameter, mm, mean (SD)	55.7 (6.8)	55.6 (5.8)	55.7 (7.2)	-0.41*	0.679

Surgical details

All patients with COPD underwent surgery using a conduit prosthesis replacing both the aortic valve and the ascending aorta, including the aortic root (7 [100.0%]), whereas 19 patients without COPD (67.9%) had a conduit prosthesis; aortic root-sparing surgery was offered to nine patients (32.1%) without COPD, including replacement of the ascending aorta with an aortic valve prosthesis, and only ascending aorta replacement in three patients (10.7%) (Table 2). 

**Table 2:** Surgical details, number (%) COPD: chronic obstructive pulmonary disease; AVR: aortic valve replacement; Categorical variables were presented as numbers and percentages n (%) and were compared using the Chi-square (χ2) or Fisher’s exact tests, when n < 5. p < 0.05 was considered significant.

	All patients	With COPD	Without COPD	Test statistic value	p-value
Conduit	26 (74.3)	7 (100.0)	19 (67.9)	3.03	0.082
Mechanical conduit	6 (17.1)	1 (14.3)	5 (17.9)	0.05	0.823
Biological conduit	20 (57.1)	6 (85.7)	14 (50.0)	2.92	0.088
Aorta prosthesis	3 (8.6)	0	3 (10.7)	0.82	0.365
AVR and aorta prosthesis	6 (17.1)	0	6 (21.4)	1.81	0.178
Mechanical AVR and aortic prosthesis	3 (8.6)	0	3 (10.7)	0.82	0.365
Biological AVR and aortic prosthesis	3 (8.6)	0	3 (10.7)	0.82	0.365

Cross-sectional measures of the thoracic aorta

As the cut-off measure of 40 mm for the aortic diameter was set for aortic dilatation, there were no significant differences in the cross-sectional measures of the thoracic aorta between patients with or without COPD. However, the diameter of the distal ascending aorta at the level of the truncus was slightly increased in patients with COPD as compared with those without COPD (36.5 [3.3] vs. 32.2 [3.8] mm, p = 0.011, respectively, Table 3).

**Table 3:** Diameters of the thoracic aorta, mean (standard deviation) COPD: chronic obstructive pulmonary disease; Continuous variables were expressed as means with standard deviation and compared using the Mann-Whitney U test. p < 0.05 was considered significant. *Test statistic values include z-values.

	All patients	With COPD	Without COPD	Test statistic value*	p-value
Number of patients n (%)	35 (100.0)	7 (100.0%)	28 (100.0)		
Aortic annulus	45.5 (6.1)	45.6 (3.8)	45.5 (6.6)	-0.29	0.773
Sinus of Valsalva	50.4 (9.9)	53.4 (8.6)	49.6 (10.2)	-1.32	0.187
Sinotubular junction	49.2 (12.2)	52.9 (10.2)	48.3 (12.7)	-1.36	0.173
Ascending aorta	51.7 (5.9)	53.6 (5.9)	51.2 (6.1)	-0.85	0.398
Distal ascending aorta	33.0 (4.0)	36.5 (3.3)	32.2 (3.8)	-2.54	0.011
Descending aorta	28.2 (3.6)	30.8 (4.5)	27.6 (3.1)	-1.75	0.080

Histological changes of the ascending aorta

**Figure 1:**
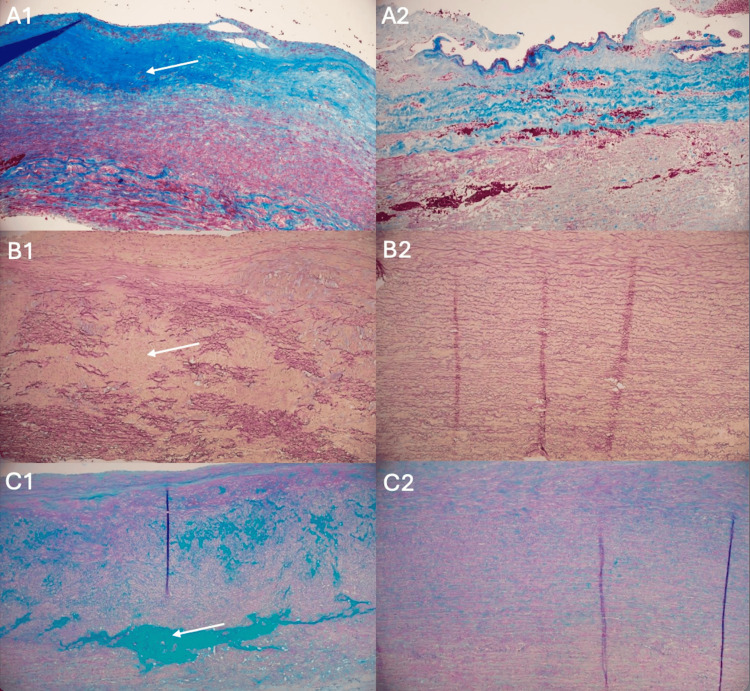
Ascending aortic histology Panel of representative ascending aorta histology showing horizontally the severe extent of A. adventitial fibrosis (arrow, Masson Trichrome, blue color 100 x magnification), B. elastic fiber loss and disorganization (arrow, Verhoeff-van Gieson, 100 x magnification), C. mucoid extracellular matrix accumulation (arrow, periodic acid-Schiff-Alcian Blue, 100 x magnification), in patients with COPD as compared to those without COPD (1 and 2, respectively).

As shown in Table 4, patients with COPD had more adventitial fibrosis, elastic fiber fragmentation and/or loss, and a higher degree of medial degeneration (0.43 [0.54] vs. 0.08 [0.27], p = 0.023; 4.43 [1.13] vs. 3.29 [1.24], p = 0.034; 2.67 [0.52] vs. 1.67 [0.73], p = 0.006, respectively) as compared with patients without COPD (Figure 1). 

**Table 4:** Ascending aortic wall degeneration, mean point score units (standard deviation) COPD: chronic obstructive pulmonary disease; Continuous variables were expressed as means with standard deviation and compared using the Mann-Whitney U test. p < 0.05 was considered significant. *Test statistic values include z-values.

	All patients	With COPD	Without COPD	Test statistic value	p-value
Number of patients	35	7	28		
Adventitial fibrosis	0.15 (0.36)	0.43 (0.54)	0.08 (0.27)	-2.27	0.023
Collagen Alteration, Medial fibrosis	0.17 (0.62)	0	0.21 (0.69)	-0.89	0.373
Elastic Fiber disorganization	0.89 (0.93)	1.43 (0.79)	0.75 (0.93)	-1.83	0.067
Elastic fiber fragmentation and/or loss	3.51 (1.29)	4.43 (1.13)	3.29 (1.24)	-2.12	0.034
Elastic fiber thinning	0.83 (1.72)	1.71 (2.14)	0.61 (1.57)	-1.66	0.097
Laminar medial collapse	0.37 (1.11)	0.43 (1.13)	0.36 (1.13)	-0.26	0.794
Medial degeneration classification	1.85 (0.80)	2.67 (0.52)	1.67 (0.73)	-2.74	0.006
Mucoid extracellular matrix accumulation	4.57 (1.15)	5.71 (1.11)	4.29 (0.98)	-2.76	0.006
Smooth muscle cell disorganization	0.49 (0.74)	0.29 (0.49)	0.54 (0.79)	-0.65	0.514
Smooth muscle cell nuclei loss	2.71 (1.62)	3.57 (1.13)	2.50 (1.67)	-1.54	0.123
Vasa vasorum medial thickening	0.23 (4.23)	0.43 (0.54)	0.18 (0.39)	-1.39	0.165

**Figure 2:**
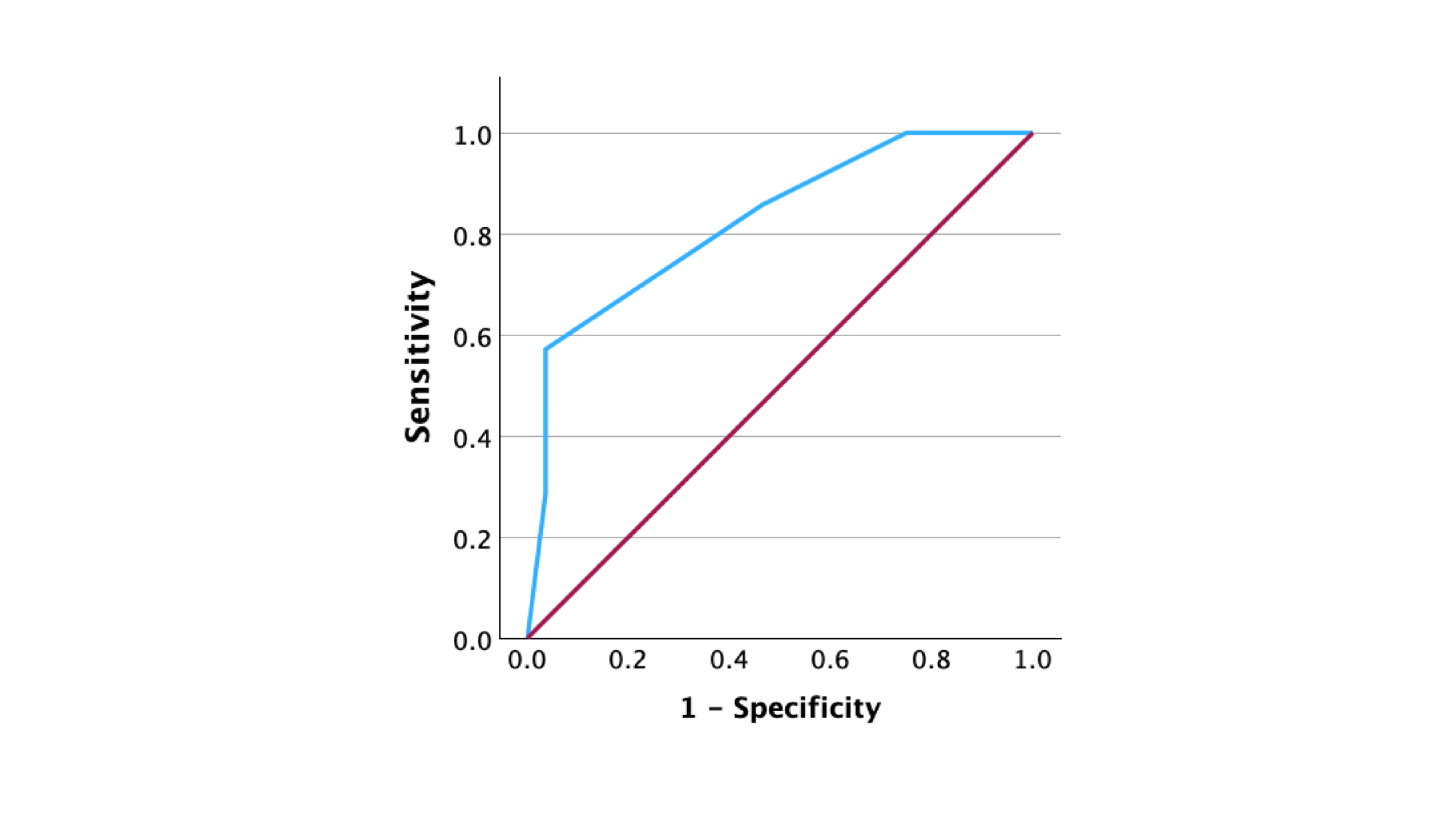
Receiver operating characteristics (ROC) curve According to ROC analysis, mucoid extracellular matrix accumulation is significantly associated with patients with COPD and ascending aortic dilatation (blue line, area under the curve [AUC], 0.827; standard error [SE], 0.091; p < .001; 95% confidence interval [CI], 0.649-1.004). Reference line (red line).

Moreover, patients with COPD had more mucoid extracellular matrix accumulation than patients without COPD (5.71 [1.11] vs. 4.29 [0.98], p = 0.006, respectively). The increased severity of medial degeneration and extracellular matrix disruption in COPD patients suggests potentially higher susceptibility to adverse aortic outcomes (area under the curve [AUC], 0.827; standard error [SE], 0.091; p < .001; 95% confidence interval [CI], 0.649-1.004, Figure 2).

## Discussion

The severity of aortic wall degeneration in patients with COPD during ascending aortic dilatation was increased compared to those without COPD. Surgical decision-making to replace the ascending aorta is based on clinical awareness of possible subsequent aortic events. The subgroup of patients with ascending aortic dilatation and COPD may benefit from close surveillance. 

COPD is a clinical entity representing various combinations of coexisting comorbidities and cardiovascular risk factors [13]. Smoking is frequently associated with COPD, and over 80% of COPD patients suffer from several symptoms [7]. COPD exhibits high rates of mortality, ranking as the third leading cause of death worldwide [14]. While patients with COPD share many traditional risk factors associated with ascending aortic dilatation, such as smoking and high blood pressure [5], the common pathogenetic risk denominator may still include distinctive features of aortic wall degeneration. 

According to histopathology evaluation, several variables of the media layer and smooth muscle cell degeneration were observed in the ascending aortic wall of patients with COPD as compared with those without COPD. Histological findings, including increased adventitial fibrosis, elastic fiber fragmentation and loss, medial degeneration, collagen alteration with medial fibrosis, and mucoid extracellular matrix accumulation, are all pertinent findings describing aortic wall degeneration [12,15]. Histopathology was obtained from the ascending aorta, and histology differed even though dilatation was statistically similar at this site statistically similar among the patient groups. It is tempting to speculate that COPD is associated with a risk of connective tissue frailty that is also reflected in increased degeneration of the medial layer of the aortic wall [9,10].

Surgery for our patients was performed according to the principle of preventing further ascending aortic dilatation. Ascending aortic dilatation and associated severity of aortic wall degeneration may increase the risk for aortic events [16]. Even though patients with COPD had increased aortic wall degeneration as compared with the rest of the patients, it is beyond the scope of this study to estimate the degree of risk of aortic events. 

Interestingly, all patients with COPD had severe aortic valve regurgitation, though aortic root dilatation did not differ among the patients. None of the patients with COPD had bicuspid aortic valves, which were present in 16 patients without COPD. We and others have previously shown that the presence of a bicuspid aortic valve per se is not associated with increased ascending aortic wall degeneration as compared with patients with tricuspid aortic valves [4,17]. Patients with COPD had very similar measures of aortic diameters, excluding a slightly larger diameter of the distal ascending aorta, as compared with patients without COPD. Aortic valve regurgitation may not solely be regarded as a cause of aortic root dilatation in our study, and a competent aortic valve may not prevent aortic dilatation [18]; there were four patients in our study with a previous aortic valve replacement. 

Limitations

This pilot retrospective cross-sectional analysis included a small number of patients; histology was obviously available only from patients undergoing surgery for ascending aortic dilatation, and longitudinal follow-up is lacking. However, the systematic measurements of the aortic diameters together with the extensive histopathologic analysis of the ascending aortic wall provide a baseline platform for identifying specific patients susceptible to ascending aortopathy. 

## Conclusions

We identified a subgroup of patients with COPD and aortic valve regurgitation who have increased ascending aortic wall degeneration as compared with patients without COPD. Patients with aortic dilatation and COPD may be associated with aortic root involvement and aortic valvular insufficiency. To attribute aortic dilatation directly to COPD, a focused study is required to determine what percentage of COPD patients who develop aortic dilatation. These findings suggest that COPD is an important risk factor for severe ascending aortic degeneration and may warrant enhanced clinical surveillance and individualized management strategies to prevent potential adverse aortic events.
